# Exclusive breastfeeding modifies the association between maternal education and child development: a cross-sectional study nested in a cohort

**DOI:** 10.1016/j.jped.2025.02.004

**Published:** 2025-04-07

**Authors:** Luiza Alves Ford, Gabriela Buccini, Amanda Castelo Saragosa, Isadora de Araújo Martins, Janaína Matos Moreira, Stela Maris Aguiar Lemos, Claudia Regina Lindgren Alves, Vivian Mara Gonçalves de Oliveira Azevedo

**Affiliations:** a Universidade Federal de Uberlândia, Uberlandia, MG, Brazil; b University of Nevada, Las Vegas, United States; c Universidade Federal de Minas Gerais, Belo Horizonte, MG, Brazil

**Keywords:** Breastfeeding, Exclusive breastfeeding, Child development, Maternal education, COVID-19

## Abstract

**Objective:**

Low maternal education is a risk factor for early childhood development (ECD), while exclusive breastfeeding (EBF) is a protective factor. This study examined the association between maternal education and ECD outcomes such as cognitive, language, and motor domains and whether EBF modifies this association in Brazil.

**Methods:**

This cross-sectional study analyzed data from a non-probabilistic sample of 12-month-old infants born during the COVID-19. Moderation analyses using the Mann-Whitney test examined the effect of EBF at 6 months (effect modifier) on the relationship between Bayley-III cognitive, language, and motor scores as well as Bayley Global Score (BGS) (outcomes) and maternal education (independent variable). The effect size (r) from the sensitivity analysis of the effect modifier was estimated.

**Results:**

A total of 269 full-term infants were evaluated. Higher maternal education was associated with better cognitive, language, and BGS (*p* < 0.00). EBF was associated with higher cognitive (*p* < 0.01), language (*p* < 0.02), and BGS (*p* < 0.00). EBF modified the effect of low maternal education (<10 years; and 10–12 years) on cognitive score and BGS. Among mothers with >10 years of education, a large effect size of EBF was observed on the BGS (*r* = 0.51), and a medium effect size was noted in the cognitive domain (*r* = 0.38).

**Conclusion:**

Higher maternal education is associated with better scores on Bayley-III domains, and EBF can modify the effect of lower maternal education on ECD in Brazil. This is the first study to identify EBF as a mechanism to protect ECD in adverse conditions such as low maternal education.

## Introduction

Approximately 43 % of infants under 5 years old who reside in low-and middle-income countries are at risk of not reaching their full developmental potential.^1^ Infants with suboptimal development across cognitive, physical, language, motor, social, and emotional skills could experience detrimental effects on their short and long-term education and income attainment, perpetuating inequalities in the cycle of poverty.^2^ The COVID-19 pandemic has deepened socioeconomic inequalities, exposing infants to adverse experiences that may have affected their development.^3^

In Brazil, the largest country in Latin America and the Caribbean, a study on early childhood development (ECD) involving 13,435 children from 0 to 59 months infants during the COVID-19 pandemic found that 39.6 % of them had below-average development compared to the national average.^4^ This study found that infants whose mothers had lower levels of education were at higher risk of having below-average development.^4^ Low maternal education has been consistently reported as a risk factor for ECD delays among diverse populations.^5^^,^^6^ On the other hand, high maternal education leads to better developmental outcomes, including cognitive, language, and motor outcomes,^2^^,^^5^ due to a more stimulating and responsive home environment.^2^ However, most studies analyzing this association have been conducted in high-income, educated, and industrialized settings.^2^^,^^5^

Maternal education has also been associated with breastfeeding practices. Analysis using data from low- and middle-income countries found that highly educated mothers had a higher prevalence of exclusive breastfeeding (EBF), whereas mothers with lower education had a worse prevalence of breastfeeding practices.^7^ Moreover, longer breastfeeding duration has been positively associated with higher intelligence scores.^8^ One possible explanation is that human milk delivers nutrients and bioactive molecules to support optimal infant growth and cognitive development.^9^ Increasing evidence has linked human milk oligosaccharides (HMOs) to brain development and better ECD milestones.^10^ Beyond nutrition, breastfeeding has been shown to strengthen maternal-infant bonding, which may lead to better cognitive and social-emotional development.^11^

In Brazil, the prevalence of EBF among infants under 6 months was 45.8 % in 2019,^12^ which is far below the World Health Organization (WHO) target of 70 % by 2030.^13^ Therefore, understanding the relationship between maternal education, breastfeeding, and ECD is critical for fostering equity in the country. Nonetheless, studies evaluating the relationship between maternal education and ECD in low and middle-income countries such as Brazil are limited,^2^^,^^5^ and to our knowledge, no previous study has attempted to investigate if EBF modifies this relationship. To close this gap, the authors aimed to examine the association between maternal education and ECD outcomes, such as cognitive, language, and motor outcomes, and whether EBF modifies this association in the context of the COVID-19 pandemic in Brazil. The authors hypothesized that EBF would positively modify the association between maternal education and ECD.

## Methods

### Study design

This cross-sectional study analyzed data from a non-probabilistic sample of infants born during the COVID-19 pandemic participating in a larger cohort study, approved by the Institutional Research Ethics Committee of the Federal University of Minas Gerais (CAAE 42269021.9.0000.5149). The authors used the Strengthening of the Reporting of Observational Studies in Epidemiology (STROBE) checklist to report this study (Supplementary Material).

### Sample and analytical sample

The analytical sample for this study included 289 infants enrolled in a broader cohort serological survey study.^14^ All children who attended the developmental assessment in person at 12 months between April and September 2022 were included in the present study. The authors excluded infants who did not complete the developmental assessment (*n* = 3), those whose mothers did not answer questions about breastfeeding at 6 months (*n* = 3), one infant from each set of twins (*n* = 2), and infants born prematurely (*n* = 12). Thus, the final analytical sample consisted of 269 infants aged 12 months.

Participants in the serological survey study were recruited from five municipalities in Southeast Brazil between April and August 2021. Newborns and their mothers' blood samples were tested for immunoglobulin G anti-N (IgG) against SARS-CoV-2. For the broader study, newborns were eligible if they were aged up to 7 days and attended public primary healthcare clinics for newborn screening accompanied by their mothers. Dyads were excluded when mothers did not respond to the clinical and sociodemographic questionnaire for any reason or were vaccinated for SARS-CoV-2 during pregnancy.^14^

Mothers were interviewed by phone at 1, 6, and 12 months after childbirth. At 12 months of the infant's age, participants were invited to participate in an in-person infant developmental assessment. Assessments were conducted by a trained health team with experience in applying the Bayley Scales of Infant and Toddler Development - Third Edition (Bayley-III).^15^ All infants had scheduled appointments, and the assessment was carried out individually in the presence of a caregiver. The administration of the Bayley-III used standardized forms and materials and lasted an average of 60 min.

### Study variables

#### Outcome variables

The outcome of this study was ECD, which was assessed when the infants were 12 months old using the Bayley-III.^15^ This scale assesses the cognitive, motor, and language skills of infants aged 1 to 42 months and is recognized for providing reliable, valid, and precise results.^15^ The *cognitive domain* assesses how infants react, explore, and solve problems, their relationship with objects, and their performance in areas such as memory, visualization, attention, and correlations between them. The *language domain* assesses receptive and expressive communication, whereas receptive communication analyzes sound recognition, understanding of words, and vocalized instructions, and expressive communication assesses pre-verbal and verbal communication using gestures, sounds, and words. The *motor domain* assesses fine and gross motor skills. The fine motor involves the use of hands and fingers to perform refined tasks and handle small objects, and the gross motor involves large body movements.^15^

The composite score for each domain ranges from 40 to 160 points. Composite scores equal to or higher than 85 were considered normal for age. For data analysis purposes, the authors also created an overall Bayley Global Score (BGS) variable by taking the arithmetic average of the cognitive, language, and motor composite scores. This provides an overall view of a child's development. The BGS was classified on the same basis as the other domains into normal or delayed results.

#### Independent variable

Maternal education was used as a socioeconomic gradient variable as it is a determinant of health and an indicator of socioeconomic status^16^ with a strong impact on child development.^6^ Maternal education was classified by years of study into three categories: <10 years of study, between 10 and 12 years of study, and >12 years of study.

#### Effect modifiers

Effect modification occurs when a third variable modifies the relationship between the independent variable and the outcome. The modifier was EBF at 6 months, defined as the exclusive intake of human milk directly from the breast, expressed, or from another source without the intake of other liquid or solid foods.^17^ For data analysis purposes, EBF was classified as yes or no.

#### Covariates

Covariables were selected based on previous empirical evidence or conceptual considerations.^18^ The covariables were:

*Infant sex* was classified as male or female based on mothers' reports.

*Maternal age* was classified as ≤ 19 years old and > 19 years old because adolescent mothers can be at increased risk for various perinatal complications and adverse birth outcomes.^19^

*Risk of maternal depression* was screened using the Patient Health Questionnaire-2 (PHQ2) based on two questions: "Over the past 2 wk, how often have you been bothered by any of the following problems: 1) having little interest or pleasure in doing things? 2) Feeling down, depressed, or hopeless?" The answer options are scored from zero to three. A total score of 3 or greater suggests a risk of maternal depression.^20^ This variable was collected during the 12-month interview.

*Daycare attendance* was reported by mothers during the 12-month interview.^21^^,^^22^ Responses were classified as yes or no.

*Stimulation activities at home* were assessed using the Family Care Indicators (FCI)^23^ from the Multiple Indicator Cluster Surveys: Cognitive Stimulation (MICS).^24^ The FCI assesses stimulation activities carried out at home with the child by someone older than 15 years during the 3 days before the interview. Activities assessed included reading, telling stories, singing, drawing, and playing outdoors. Stimulation was considered satisfactory if the child participated in ≥ 4 activities and unsatisfactory if <4.^18^

### Data analyses

Data was collected and extracted via GoogleForms® and exported to Epi Info software version 7.2.5.0 and software R version 4.4.0 for data analysis.

Descriptive analysis explored the frequency of categorical variables and central tendency and dispersion measures of continuous variables. The normality of the score distributions in the Bayley-III domains was examined using the Shapiro-Wilk test, indicating that the distribution was non-normal. Bivariate analysis using the Kruskal-Wallis's test explored the association between maternal and child characteristics across the Bayley-III scores.

To test the hypothesis, the authors performed a moderation analysis using the Mann-Whitney test to examine the effect of EBF on the relationship between Bayley-III scores and maternal education levels.

A sensitivity analysis was conducted utilizing violin plots to illustrate the distribution of composite Bayley-III scores across each domain (cognitive, language and motor) and overall BGS between the groups (with and without EBF) stratified by maternal education. The effect size (r) of the EBF pattern on Bayley-III scores stratified by maternal education was also calculated using AI-Therapy Statistics. The significance level was considered 5 % in all analyses.

## Results

### Descriptive analysis

A total of 269 infants were evaluated at 12 months of age. The sample predominantly consisted of adult mothers (*n* = 253, 95.47 %) who had studied for 10–12 years (*n* = 156, 58 %) and were not at risk of being depressed (*n* = 229, 85.13 %). Most of the infants were male (*n* = 149, 55 %) and were not attending daycare centers (*n* = 230, 85.50 %). A little over a third of the infants were EBF at six months (*n* = 102, 37.92 %). Regarding stimulation activities at home, 60.22 % of the children participated in at least four activities three days before the interview (*n* = 162). According to Bayley-III, 2.97 % of the infants had delays in the cognitive domain (*n* = 8), 16.73 % in the language (*n* = 45), 7.06 % in the motor domain (*n* = 19), and 4.83 % of the infants were globally delayed (*n* = 13) (Table 1).

**Table 1:** Characteristics of the mother-infant pairs (*n* = 269).

Maternal characteristics	n (%)
Age	
≤19 years	12 (4.53)
>19 years	253 (95.47)
Missing data	4 (1.49)
Education	
<10 years	28 (10.41)
10–12 years	156 (57.99)
>12 years	85 (31.60)
Risk of Maternal Depression	
Yes	40 (14.87 %)
No	229 (85.13 %)

**Infant characteristics**	
Sex	
Female	120 (44.61 %)
Male	149 (55.39 %)
Exclusive Breastfeeding at 6 months	
Yes	102 (37.92 %)
No	167(62.08 %)
Stimulation activities at home^a^	
<4 activities	107 (39.78)
≥4 activities	162 (60.22)
Daycare attendance	
Yes	39 (14.5)
No	230 (85.5)
Results of child development according to the Bayley-III	
Cognitive Domain	
Delayed	8 (2.97 %)
Normal	261 (97.03 %)
Language Domain	
Delayed	45 (16.73 %)
Normal	224 (83.27 %)
Motor Domain	
Delayed	19 (7.06 %)
Normal	250 (92.94 %)
Bayley Global Score (BGS)^b^	
Delayed	13 (4.83 %)
Normal	256 (95.17 %)

a Family Care Indicators (FCI) indicates the number of stimulation activities, such as sing, read a book, tell stories and play outside, someone older than 15 years done with the child in the last 3 days.

b Arithmetic average of the cognitive, language, and motor composite scores, providing an overall view of a child's development, classified on the same basis as the other domains into normal or delayed results.

### Bivariate analysis

Higher maternal education (> 12 years) was associated with higher means of infant cognitive, language, and overall BGS scores compared to mothers with 10–12 years or < 10 years of education. EBF at 6 months was associated with higher cognitive, language, and overall BGS scores compared to infants, not EBF at 6 months (Table 2). None of the other covariates were associated with any Bayley-III scores.

**Table 2:** Bivariate analysis between maternal and infant characteristics and Bayley-III composite scores and BGS.

Bayley-III Scores									
		COGNITIVE scores		LANGUAGE scores		MOTOR scores		BAYLEY GLOBAL scores (BGS)^b^	
		Median (IQ 25–75)	*p*-value	Median (IQ 25–75)	*p*-value	Median (IQ 25–75)	*p*-value	Median (IQ 25–75)	*p*-value
Maternal Characteristics									
Maternal Education (Years)	< 10	110.00 (100.00–115.00)	**0.00**	97.00 (83.00–101.50)	**0.00**	101.50 (91.00–107.00)	0.83	101.00 (96.50–105.17)	**0.00**
	10–12	110.00 (105.00–120.00)		97.00 (89.00–103.00)		100.00 (91.00–107.00)		103.33 (96.50–108.50)	
	> 12	115.00 (110.00–120.00)		103.00 (94.00–112.00)		103.00 (91.00–110.00)		106.67 (101.33–111.33)	
Maternal Age (Years)	≤ 19	110.00 (105.00–117.50)	0.74	98.50 (92.50–101.50)	0.73	101.50 (89.50–107.00)	0.84	100.67 (98.00–105.66)	0.64
	> 19	110.00 (105.00–120.00)		100.00 (89.00–106.00)		100.00 (91.00–110.00)		104.33 (97.33–109.00)	
Risk of Maternal Depression	Yes	110.00 (100.00–120.00)	0.31	97.00 (89.00–106.00)	0.14	98.50 (88.00–103.00)	0.11	102.17 (94.17–106.83)	0.11
	No	110.00 (105.00–120.00)		100.00 (89.00–106.00)		100.00 (91.00–110.00)		104.33 (97.67–109.00)	
Infant Characteristics									
Infant Sex	Female	115.00 (105.00–120.00)	0.09	100.00 (90.00–106.00)	0.18	100.00 (94.00–111.00)	0.09	105.00 (98.83–110.17)	0.06
	Male	110.00 (100.00–120.00)		97.00 (89.00–106.00)		100.00 (91.00–107.00)		103.67 (97.33–108.33)	
Exclusive Breastfeeding at 6 months	Yes	115.00 (110.00–120.00)	**0.01**	100.00 (91.00–109.00)	**0.02**	103.00 (91.00–110.00)	0.38	106.00 (99.67–110.00)	**0.00**
	No	110.00 (100.00–120.00)		97.00 (89.00–106.00)		100.00 (91.00–107.00)		102.67 (96.00–108.33)	
Daycare attendance	Yes No	111.97 (110.00–120.00) 110.39 (105.00–120.00)	0.82	98.97 (89.00–106.00) 97.03 (89.00–106.00)	0.52	98.76 (91.00–103.00) 100.01 (91.00–110.00)	0.43	103.24 (98.67–107.33) 102.48 (97.33–109.00)	0.88
Stimulation activities at home^a^	< 4 ≥ 4	109.20 (100.00–120.00) 111.56 (105.00–120.00)	0.08	95.67 (89.00–106.00) 98.39 (89.00–106.00)	0.16	96.70 (91.00–110.00) 99.92 (94.00–107.00)	0.83	101.52 (96.00–108.00) 103.29 (98.00–109.50)	0.14

a Family Care Indicators (FCI) indicate the number of stimulation activities, such as singing, reading a book, telling stories, and playing outside, someone older than 15 years done with the child in the last 3 days

b Arithmetic average of the cognitive, language, and motor composite scores, providing an overall view of a child's development.

### Moderation analysis

EBF at 6 months modified the effect of maternal education and BGS scores for mothers less schooled. Children from mothers who studied for <10 years and were EBF had higher cognitive scores (*p* = 0.04) and BGS (*p* = 0.00) than those not EBF. Children from mothers who studied for 10–12 years and were EBF had higher scores in cognitive domain (*p* = 0.04) and BGS (*p* = 0.05) than their counterparts. These effects were not observed for children from mothers who studied for >12 years in any developmental domains (Table 3).

**Table 3:** Association between maternal education and the Bayley-III composite scores and BGS stratified by exclusive breastfeeding at 6 months.

		Bayley-III Scores											
		COGNITIVE scores			LANGUAGE scores			MOTOR scores			BAYLEY GLOBAL scores (BGS)^b^		
Maternal Education	EBF at 6 months	Median (Min-Max)	*p*-value	Effect size (r)	Median (Min-Max)	*p*-value	Effect size (r)	Median (Min-Max)	*p*-value	Effect size (r)	Median (Min-Max)	*p*-value	Effect size (r)
< 10 years (*n* = 28)	Yes (*n* = 8)	112.50 (100.00–120.00)	**0.04**^a^	0.38	98.50 (83.00–124.00)	0.08	0.29	105.00 (88.00–127.00)	0.21	0.24	104.83 (98.33–120.67)	**0.00**^a^	0.51
	No (*n* = 20)	102.50 (660.00–125.00)			94.00 (65.00–106.00)			98.50 (79.00–115.00)			98.67 (68.00–107.67)		
10–12 years (*n* = 156)	Yes (*n* = 57)	110.00 (70.00–135.00)	**0.04**^a^	0.17	100.00 (56.00–118.00)	0.07	0.15	100.00 (67.00–127.00)	0.41	0.07	105.33 (64.33–124.67)	**0.05**^a^	0.15
	No (*n* = 99)	110.00 (75.00–130.00)			97.00 (53.00–118.00)			100.00 (70.00–127.00)			102.00 (66.0–117.66)		
> 12 years (*n* = 85)	Yes (*n* = 37)	115.00 (75.00–130.00)	0.71	0.04	103.00 (62.00–127.00)	0.84	0.20	103.00 (58.00–124.00)	0.75	0.03	106.00 (65.00–122.00)	0.91	0.01
	No (*n* = 48)	115.00 (95.00–135.00)			103.00 (50.00–147.00)			101.50 (79.00–121.00)			106.67 (82.67–126.33)		

EBF, Exclusive breastfeeding at 6 months.

a *p*-value ≤ 0.05.

b Arithmetic average of the cognitive, language, and motor composite scores, providing an overall view of a child's development.

### Sensitivity analysis

**Figure 1:**
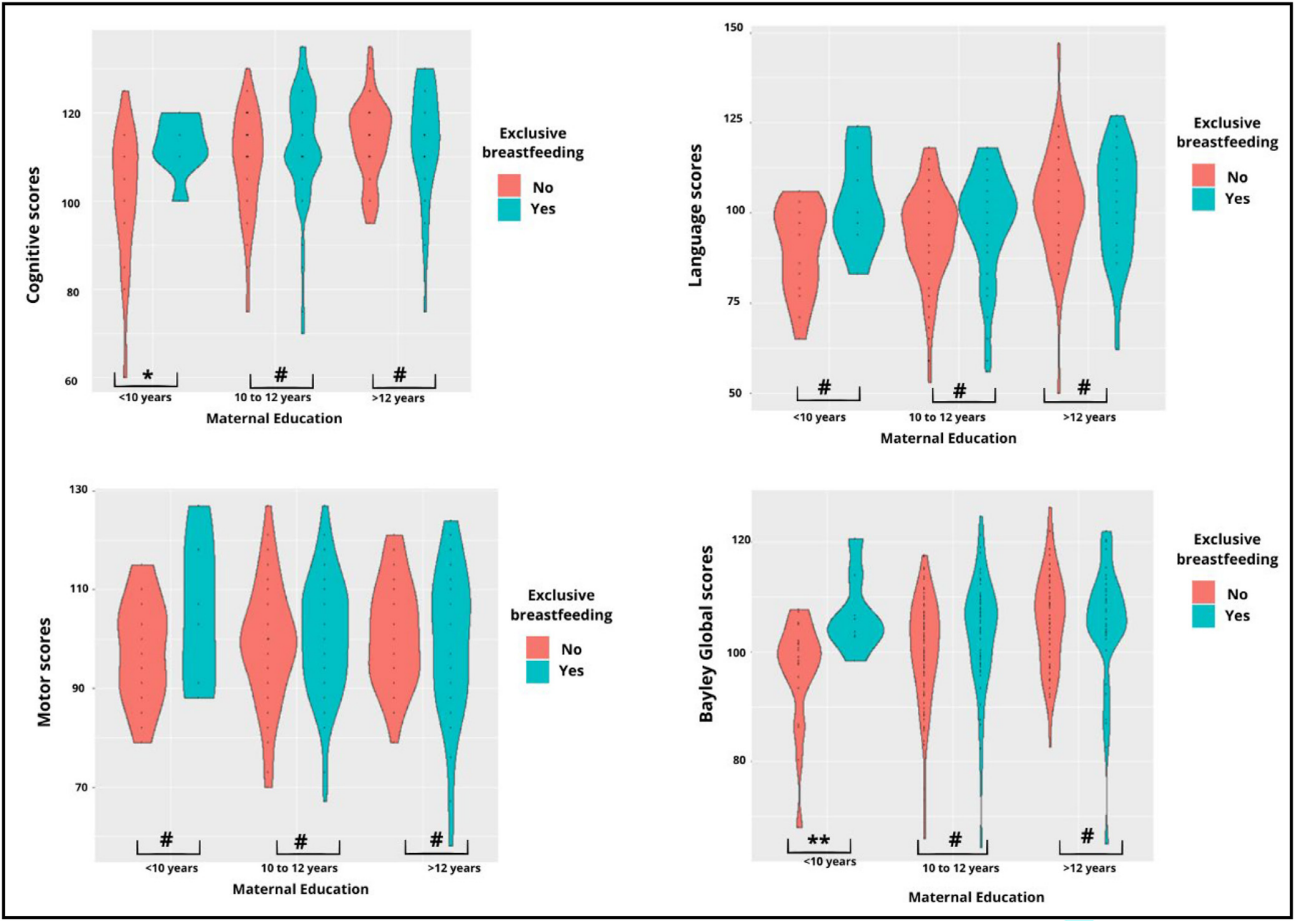
Distribution of Bayley-III composite scores an BGS comparing children with and without EBF, stratified by maternal education. Effect size *r = 0.38 (medium); ^⁎⁎^*r* = 0.51(large); ^#^*r* < 0.30 (small or very small); ^⁎⁎⁎^Arithmetic average of the cognitive, language, and motor composite scores, providing an overall view of a child's development.

Figure 1 depicts the violin plots of the distribution of composite Bayley-III scores across each domain (cognitive, language, motor) and BGS, comparing groups with and without EBF, stratified by maternal education. Among mothers with <10 years of education, a large effect size of EBF was observed on the overall BGS (*r* = 0.51), while a medium effect size was noted in the cognitive domain (*r* = 0.38) (Figure 1). The effect size for the other domains was not significant (small or very small).

## Discussion

The present study found that higher maternal education (> 12 years) and EBF at 6 months were associated with better performance in cognitive and language domains, and higher BGS. These findings were innovated by documenting the moderator effect of EBF at 6 months on the relationship between maternal education and ECD, specifically in the cognitive domain and child global development for less schooled mothers (< 12 years). To our knowledge, this is the first study to investigate the moderator effect of EBF on the relationship between maternal education and ECD. These findings are important as they identify EBF as a mechanism to protect ECD in adverse conditions such as low maternal education.

Higher maternal education was associated with higher cognitive, language, motor, and overall scores, as described in prior studies worldwide, indicating lower risk for development.^2^^,^^6^ Furthermore, these findings demonstrated that EBF at 6 months can buffer the negative effect of lower maternal education on cognitive scores. Previous studies indicate that the positive association between breastfeeding and cognitive development is due to nutrients available through breast milk.^10^^,^^25^^,^^26^ For example, HMOs are among the most important factors in forming the intestinal microbiota and have a critical role in brain maturation, contributing to better cognitive development in early childhood.^27^^,^^10^ Another well-known component of human milk is long-chain polyunsaturated fatty acids (LCPUFAs), including docosahexaenoic acid (DHA) and arachidonic acid (AA), which are nutrients that also assist brain development.^25^^,^^26^ Studies showed that concentrations of DHA and AA were lower when children were formula-fed^25^^,^^26^ and those breastfed children had higher cognitive scores than formula-fed children.^25^ Therefore, it is plausible that although the children's mothers had low education, the nutrients in their human milk helped their infants' brain development and, in turn, helped with their cognitive development.

Increased maternal-infant bonding due to breastfeeding could be another explanation for the positive association between breastfeeding and cognitive development.^11^^,^^25^ Studies have shown that children with strong maternal-infant bonding have increased cognitive development.^11^^,^^25^ It has been found that the infant's overall brain development can be affected by their bond with their mothers.^11^^,^^28^ Therefore, it is plausible to assume that skin-to-skin contact and interaction during breastfeeding may increase the infant's bond with the mother, increasing their development.^11^^,^^25^

The present study also found that EBF modified the effect of maternal education on overall child development, neutralizing the negative effect of low maternal education on BGS. The BGS represents an overall view of a child's development by summing and averaging the cognitive, language, and motor scores. It is worth noting that children from less-schooled mothers performed better in all domains when they were EBF at 6 months, although the differences between groups were not significant in the language and motor domains. Thus, it makes sense that the overall BGS scores also increased in the EBF group. Despite only the cognitive scores significantly increased due to EBF, other studies have shown that breastfeeding and human milk, in general, promote infant growth and development.^9^^,^^11^

The World Health Organization (WHO) recommends EBF for the first six months of life, with continued breastfeeding until at least two years of age,^13^ given the recognized benefits for infants and maternal health.^13^^,^^29^ Despite these benefits, global rates of EBF remain below the WHO's 70 % target by 2030.^13^ The prevalence of EBF at six months in the present study (37.9 %) was slightly lower than the most recent national prevalence (45.8 %).^12^^,^^13^ This difference is expected, as this study measured EBF at six months, whereas the national prevalence includes all infants under six months. Additionally, data collection occurred during the COVID-19 pandemic, which disrupted daily life and impacted breastfeeding practices.^30^^,^^31^ A higher risk of EBF discontinuation was observed among those with less workplace flexibility who had to continue working outside the home.^31^ Furthermore, increased anxiety and stress levels during COVID-19 have been linked with lower EBF rates.^32^^,^^33^ These factors may explain the differences in EBF prevalence between the present findings and national data.

Prior research shows that mothers with lower levels of education or who work outside the home, especially without maternity leave, are more likely to discontinue EBF.^34^ In the past decade, Brazil has implemented various initiatives to promote, protect, and support breastfeeding, such as the Baby-Friendly Hospital Initiative (BFHI) and the Brazilian Strategy for Breastfeeding and Complementary Feeding Promotion (EAAB) in primary care.^35^ Building on these efforts, the present findings can further inform the development of new policies and reinforce exclusive breastfeeding as a key strategy for supporting infant development during the first year of life, a crucial period for learning, growth, and long-term health.^29^

Some strengths and limitations should be considered when interpreting these findings. First, this was a cross-sectional study, and the authors cannot infer causation. Despite this, the present study provides a baseline understanding of EBF's effect on ECD and how it can modify negative factors that affect ECD. Moreover, the authors did not adjust the analyses for confounding factors, such as parental stress. However, the results showed that the risk of maternal depression and FCI did not affect the results despite the well-established potential of home stimulation on development in the first year of life.^18^ Additionally, the authors did not perform an *a priori* power analysis to determine the sample size necessary for the analysis presented, which may limit the generalizability of the present findings. Furthermore, a gold standard development scale was used to assess neurodevelopment in several areas with reliable and accurate results.

In conclusion, this study showed that EBF positively modified the association between maternal education and ECD, demonstrating a protective effect on the development of children in their first year of life at a lower maternal education level. These results reinforce the need for policies and actions that ensure EBF for up to 6 months, as recommended by the WHO, to mitigate the effects of low maternal education on ECD and other known benefits. The authors recommend that further studies be conducted to confirm the causal relationship between EBF and the association between maternal education and ECD.

## Funding

CNPq (40920520214 – Chamada Universal) and CAPES.

## Conflicts of interest

The authors declare no conflicts of interest.

## Appendix Supplementary materials

Image, application 1
